# Application of Robust, Packaged Long-Period Fiber Grating for Strain Measurement

**DOI:** 10.3390/mi7080129

**Published:** 2016-07-26

**Authors:** Ke-Ping Ma, Chao-Wei Wu, Tso-Sheng Hsieh, Min-Yuan Hsieh, Chia-Chin Chiang

**Affiliations:** Department of Mechanical Engineering, National Kaohsiung University of Applied Sciences, Kaohsiung 807, Taiwan; 1103403103@gm.kuas.edu.tw (K.-P.M.); cafa95011@gmail.com (C.-W.W.); srcx2s904@gmail.com (T.-S.H.); a01@phmhs.phc.edu.tw (M.-Y.H.)

**Keywords:** optical fiber sensor, long-period fiber grating, strain sensor

## Abstract

This paper proposes an optical fiber strain sensor based on packaged long-period fiber gratings (PLPFG) which is fabricated by the micro-electromechanical systems (MEMS) process and packaged with poly-dimethylsiloxane (PDMS) polymer materials. The optical fiber sensor packaged with PDMS improves robustness effectively. The proposed PLPFG sensors have periods of 610, 650, 660 μm and fiber diameter of 48, 60, 72 μm, respectively. The resonance dip of the PLPFG grows when a strain loaded onto the sensor. The results show that the largest strain sensitivity of the PLPFG strain sensor was −0.0652 dB/με from 0–1200 με and the linearity (*R*^2^) was 0.9812. Accordingly, the proposed PLPFG sensor has good potential for high-sensitivity strain sensing applications.

## 1. Introduction

Recently, the long-period fiber grating (LPFG) has been widely used for a number of applications in the sensing field. This widespread usage is owed to the fact that LPFG has a variety of unique advantages, including light weight, a small diameter, a broad measuring range, and resistance to Electromagnetic interference. Therefore, LPFG has been applied in making measurements of various physical quantities, including strain [1], displacement, torsion [2], pressure, refraction index [3], magnetic fields [4], and vibration. Among these various types of measurements, measurements of strain constitute an integral part of the engineering field. The present study proposes a packaged LPFG (PLPFG) strain sensor with a periodic photoresist grating structure and a polydimethylsiloxane (PDMS) coating. When a strain is applied to the fiber grating, the periodic grating structure will deform, in turn, causing a periodic change in the refractive index.

In 2008, Caldas et al. [5] utilized the arc discharge method to fabricate LPFG based on B/Ge co-doped fiber. The strain sensitivity of their LPFG was shown to reach up to 0.172 pm/με at tensile strains ranging from 167 to 2000 με. In 2012, Xin et al. [6] presented a strain sensing system based on LPFG that was fabricated using the CO_2_ laser method. For that study, the sensitivity of the sensor was shown to reach up to 0.75 dB/N.

In 2014, Zhong et al. [7] utilized the pressure-assisted CO_2_ laser beam scanning technique to fabricate inflated LPFG (I-LPFG) for strain measurement. The authors then conducted an experiment in which tensile strain applied to the sensor. The results showed that the sensitivity of the I-LPFG strain sensor was −5.62 pm/με. In 2015, Wang et al. [8] proposed phase-shifted LPFG fabricated by CO_2_ laser for strain measurement. Three kinds of phase-shifted LPGs with phase shifts of 0, 0.5π, and 1.0π respectively, were fabricated. The strain measurement accuracies for these LPGs were about ±30 με. In 2016, Zeng et al. [9] proposed tapered and CO_2_ laser notched LPFGs for the simultaneous measurement of strain and temperature. The results indicated that before cascading, the CO_2_-LPFG had a strain sensitivity of 1.7 pm/με and the tapered LPFG had a strain sensitivity of −1.4 pm/με. In this study, we proposed a low cost, robust PLPFG strain sensor. The PLPFG strain sensor consists of a small cladding optical fiber, periodic thick photoresist structure and PDMS polymer as the packaged material [10,11,12,13,14,15]. Unpackaged long period fiber grating strain sensors are susceptible to being influenced by the adhesive used, which can cause their strain measurement results to be inaccurate. However, the PDMS used in the proposed sensor can prevent the adhesive from penetrating into the optical fiber grating during the surface bonding process, giving it greater accuracy in strain monitoring while also allowing it to adhere conveniently to the device being tested when performing measurements. Therefore, the PLPFG strain sensor can improve the strength and stability of the grating. Therefore, the proposed PLPFG strain sensor has considerable structural integrity and convenience.

## 2. Working Principle of the PLPFG

LPFG consists of periodic refractive index variations with periods of 100–1000 μm. When light is being transmitted in LPFG, the grating will generate a resonant dip in the spectrum based on the coupled mode theory [10]. The proposed PLPFG is made of etched optical fiber sandwiched by a periodic composite soft (PDMS) and hard (SU-8) polymer structure. When the loading is applied on the PLPFG, the strain difference increases according to the periodic soft (PDMS) and hard (SU-8) cross-section area. A loading applied on the PLPFG will cause the periodic structures to become large, thereby affecting the AC coupling coefficient $K_{co-cl}^{ac}$. The resulting periodic refractive index variance in the optical fiber grating will then form a characteristic LPFG spectrum. Therefore, strain loading can effectively be used to tune the PLPFG transmission loss. When strain is applied to this PLPFG, the PLPFG reflects the strain in the form of loss variation of the transmission loss. Hence, we can measure the strain variation by monitoring the resonant dip loss.

According to the coupled mode theory [16] for LPFG, the transmission loss of a PLPFG has a cosine-squared relationship and is defined as follows:

(1) $$T=\cos^{2}(K_{co-cl}^{ac}L)$$

where *L* indicates the length of the PLPFG, the $K_{co-cl}^{ac}$ is the AC component of the coupling coefficient. The transmission loss (*T*) is a function of $K_{co-cl}^{ac}$, which is related to the amplitude of the refractive index change caused by applied strain field. Therefore, the loss can be changed by external strain loading. Using Equation (1), the applied strain can be measured by monitoring the transmission loss of the PLPFG.

## 3. Materials and Methods

### 3.1. Process and Fabrication of PLPFG

The lithography and etching processes were adopted as the process for fabricating PLPFG strain sensor. Before starting fabrication process, copper was deposited via sputtering on the 4-inch wafer. The single mode optical fiber length of 30 cm and stripped 3 cm used to etched region, the optical fiber was etched from 125 to 48, 60, 72 μm, respectively, with buffer oxide etching (BOE) solution at a temperature of 40 °C. In the beginning, the negative photoresist SU-8 3050 was spin on the wafer as the base layer. Then, the coated wafer was placed on a heating plate with to carry out a soft bake (SB) operation. After the SB operation, the exposure machine was performed using an ABM alignment system ATI-1248 (365 nm, 210 mW/cm^2^). The exposure machine was used to carry out an exposure operation with a plastic mask. The plastic mask was designed with periods of 610, 650, and 660 μm to achieve a resonant wavelength with a transmission dip about 1550 nm. Then, the wafer was placed on a hot plate to carry out a post-exposure baking (PEB) operation. Finally, after the above operations were completed, the photoresist was immersed in a developing solution to obtain the designed bottom periodic structure.

Next, the patterned SU-8 3050 photoresist structure (37.5 μm) was first spin-coated onto the surface of the wafer, and then the etched optical fiber was pasted onto the patterned SU-8 structure. The same lithography procedures are then repeated to produce the optical fiber grating device for sensor package process. Finally, the prepared PDMS was spin-coated onto the surface of the wafer to form a thickness of about 162.5 μm, and then the device was placed in an oven for curing process (temperature of 100 °C for 1 h). Finally, the package process of the PLPFG was completed.

**Figure 1:**
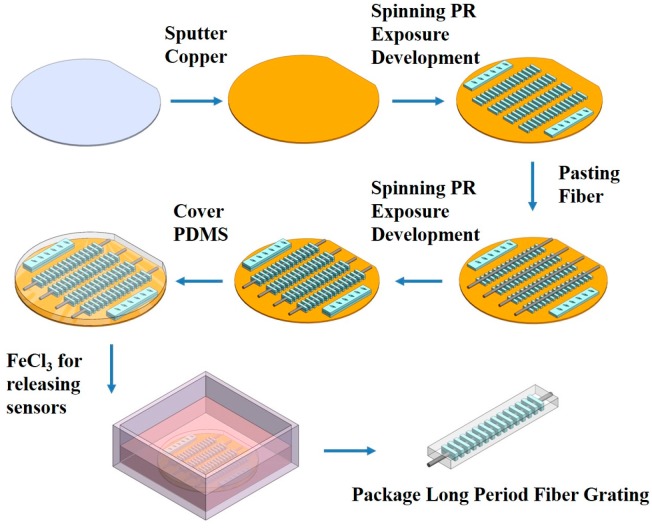
The production process of the packaged long-period fiber gratings (PLPFG) strain sensor.

The PLPFG on the wafer was then immersed in a ferric chloride solution for the releasing process for separating from the wafer. Through the proposed photoresist mass production process in Figure 1, we can obtain 12 PLPFG strain sensors per wafer.

### 3.2. Setup for PLPFG Tensile Experiment

**Figure 2:**
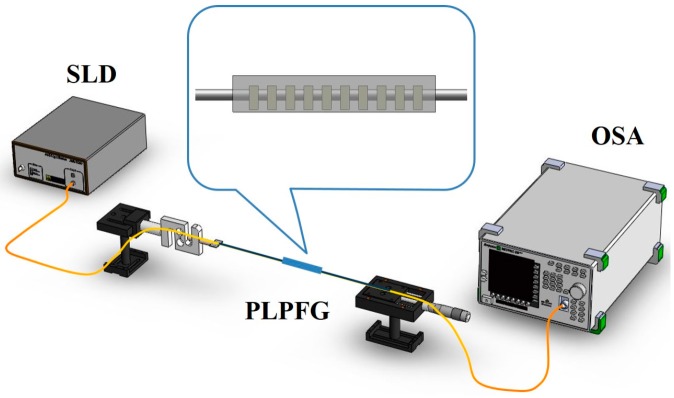
PLPFG tensile test setup diagram.

A tensile test was then conducted for strain calibration of the PLPFG sensors. The PLPFG was placed on a precision stage and then applied the strain loading. The experimental setup consists of a broadband light source, an optical spectrum analyzer (OSA), a computer, load cells, and a precision stage shown in Figure 2. In the tensile test for the PLPFG, as external axial tensile strain was applied, the strain increased accordingly in different sections of the PLPFG. Then, the spectra of the PLPFG were deformed owing to the applied the strain loading and the transmission loss of the PLPFG could similarly be changed with strain in sensing applications.

## 4. Results and Discussion

**Figure 3:**
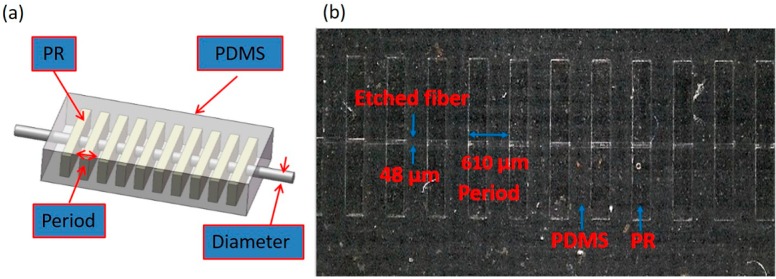
(**a**) Schematics of the PLPFG; (**b**) Optical microscopy image of the PLPFG.

In this paper, a low cost and robust PLPFG sensor was fabricated using the photolithography process. The structure of this PLPFG strain sensor was the etched optical fiber embedded in two layers of the thick photoresist (SU-8 3050) and with PDMS package. The periodic rectangular structure is the SU-8 photoresist grating in the Figure 3. The schematics and scanning electron microscope (SEM) photograph of the etched region of the PLPFG are shown in Figure 3.

**Figure 4:**
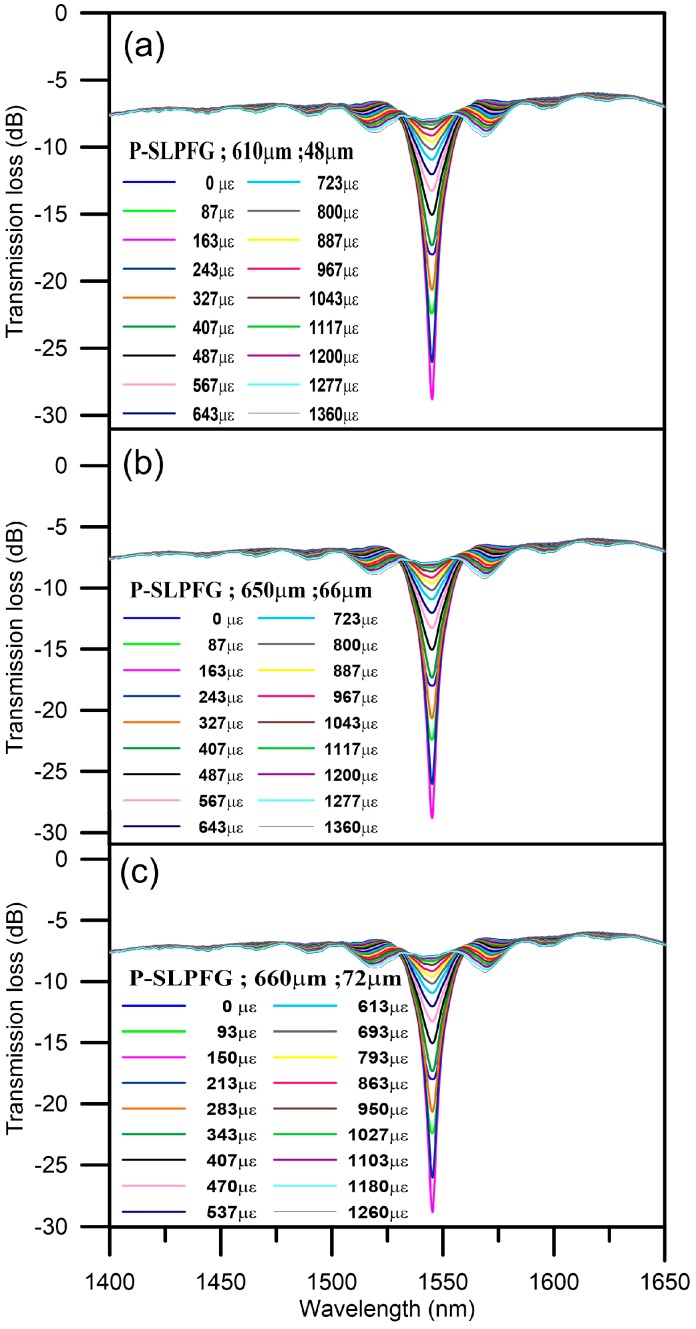
Spectra of the PLPFG under various tensile loadings: (**a**) period 610 μm, diameter 48 μm, (**b**) period 650 μm, diameter 66 μm, and (**c**) period 660 μm, diameter 72 μm.

The tensile calibration of the PLPFG strain sensor was carried out by using different strain loading (range: 0–1200 με) to vary the strain field and thereby cause the spectra to be deformed. Figure 4a–c shows the development of transmission loss by observing the spectra of the PLPFG with different loading increments. The spectra of three PLPFG sensors were with periods of, respectively, 610, 650, and 660 μm, and with differing diameters of, respectively, 48, 66, and 72 μm, in different strain loading. The spectra of the PLPFG were deformed under increasing strain loadings. Before loading, the resonant dip is weak in the spectra and the resonant dip of PLPFG is increasing with loadings and the wavelength of PLPFG slightly blue shifts during tensile test. As the loading was increased, the resonance dip was deepened according to Equation (1), which indicates that the $K_{co-cl}^{ac}$ (AC the coupling coefficient) was effectively changed with strain field. With increased strain loadings the resonance dip was deepened. The maximum transmission loss of the PLPFG were −28.68 dB at a wavelength of 1545.0 nm (Period: 610 μm, Diameter: 48 μm), −25.94 dB at a wavelength of 1552.0 nm (Period: 650 μm, Diameter: 66 μm), −21.75 dB at a wavelength of 1549.0 nm (Period: 660 μm, Diameter: 72 μm), under loading.

**Figure 5:**
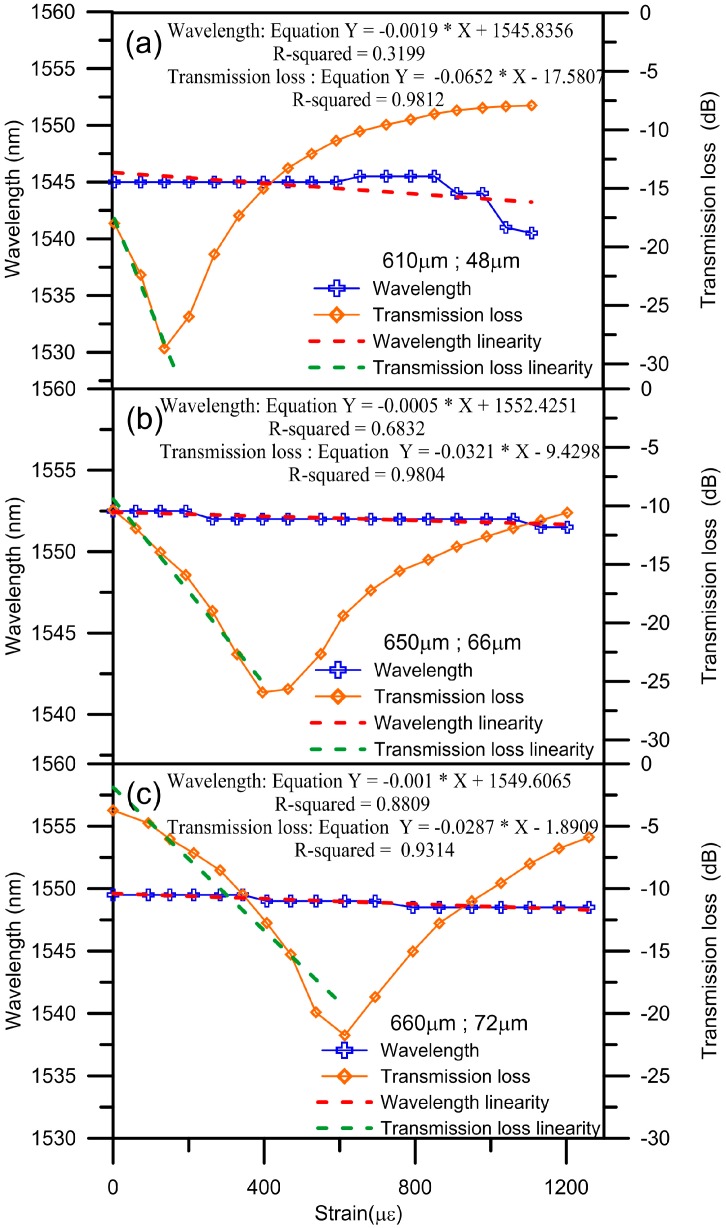
Transmission loss versus wavelength of the PLPFG under various strain loadings: (**a**) period 610 μm, diameter 48 μm, (**b**) period 650 μm, diameter 66 μm, and (**c**) period 660 μm, diameter 72 μm.

Figure 5a–c shows the diagram of transmission loss versus wavelength of the PLPFG under various strain loadings. In Figure 5, the resonant dips of the PLPFG signify growth up to the maximum transmission loss with loading. The tensile loading and transmission loss form a quadratic polynomial relationship and it can be explained using Equation (1). The sensitivities of the PLPFG were 0.0652 dB/με, −0.0321 dB/με, 0.0287 dB/με in Figure 5, respectively. The *R*^2^ value reached 0.9812, 0.9804, 0.9314, respectively. According to the tensile test results of the different PLPFG sensors with different diameters, the largest strain sensitivity was −0.0652 dB/με when the diameter was 48 μm. Hence, the sensitivity of a PLPFG strain sensor can be improved by reducing the diameter of the PLPFG used to make it.

## 5. Conclusions

The MEMS process was utilized to fabricate the PLPFG with PDMS packaging in this paper. When axial strain loading was applied to the PLPFG strain sensor, the PLPFG spectra were changed with transmission loss. Tensile strain calibration experiment results for the sensor were compared with strain gauge to confirm that the PLPFG strain sensor can indeed be used for sensing applications. The experimental results showed that the generated the largest strain sensitivity of the PLPFG strain sensor was −0.0652 dB/με and that the *R*^2^ value reached 0.9812. With the PDMS packaging, the PLPFG was effectively more robust for use as a strain sensor. The proposed sensor can be surface bonded onto an object for strain measurement. Therefore, the proposed PLPFG has good potential for use in strain sensing applications.
